# Preparation of Multilayered Core–Shell Fe_3_O_4_-SnO_2_-C Nanoparticles via Polymeric/Silane–Amino Functionalization

**DOI:** 10.3390/nano11112877

**Published:** 2021-10-28

**Authors:** Jae Uk Hur, Gye Seok An, Sung-Churl Choi

**Affiliations:** 1 Division of Materials Science and Engineering, Hanyang University, 222 Wangsimni-ro, Seongdong-gu, Seoul 04763, Korea; hjaeuk92@hanyang.ac.kr; 2 Department of Advanced Materials Engineering, Kyonggi University, 154-42 Gwanggyosan-ro, Yeongtong-gu, Suwon-si 16227, Korea

**Keywords:** Fe_3_O_4_-SnO_2_-C, core–shell, surface modification, amino functionalization, carbonization

## Abstract

Multilayered core–shell Fe_3_O_4_-SnO_2_-C nanoparticles were prepared via surface treatment and carbonization at atmospheric pressure. Fe_3_O_4_-SnO_2_ nanoparticles were prepared by the carboxylation of the pivotal particles (Fe_3_O_4_) with an anionic surfactant to immobilize SnO_2_ nanoparticles. A method was proposed to externally surround hydrophilic carbon with amine-forming materials, polyethyleneimine (PEI), and (3-Aminopropyl) triethoxysilane (APTES). The synthesis strategy was based on the electrostatic bonding of the introduced amine group with the hydroxyl group on the carbon precursor and the carbonization of the coating layer by the catalytic reaction of sulfuric acid.

## 1. Introduction

Owing to their unique electrochemical and magnetic properties, magnetite (Fe_3_O_4_) nanoparticles have gained significant attention for application in various fields, including biomedical fields [1], catalysis [2], resistive switching memory [3], energy storage [4], and electromagnetic interference (EMI) shielding [5]. However, they suffer from low chemical stability and agglomeration owing to their relatively high surface energy [6,7]. Therefore, to overcome these limitations of Fe_3_O_4_, complementary and related maintenance functions are implemented through the formation of composites with various functional groups [8,9,10] and organic/inorganic materials [7,11,12,13,14,15].

Among the various materials that are used for forming composites with Fe_3_O_4_, tin dioxide (SnO_2_), an n-type semiconductor with a wide bandgap (Eg = 3.6 eV at 300 K), has been extensively investigated [16]. Owing to its unique characteristics, SnO_2_ can stabilize the electronic, thermal, and chemical properties of Fe_3_O_4_ through the proximity effect and equilibration of potentials [17,18,19], and various efforts have been made to realize the electrochemical applications of the composites of Fe_3_O_4_ and SnO_2_. However, Fe_3_O_4_-SnO_2_ composite materials show poor electrical conductivity, which limits their applications. Therefore, it is necessary to combine these composites with high-conductivity materials, such as precious metals and carbon materials, to enhance their conductivity [20,21].

In particular, numerous studies have been carried out on the preparation of composites of Fe_3_O_4_-SnO_2_ particles with carbon-based materials to improve their conductivity and performance for application in various fields. Wang et al. [22] prepared a composite obtained by combining core–shell Fe_3_O_4_@SnO_2_ with reduced graphene oxide (rGO), a carbon-based oxide, for application in EMI shielding. Iron oxide with high magnetic permeability and low complex permittivity improved the dielectric properties of the composite, thus improving its EM wave absorption performance. Furthermore, the combination of rGO (with high electrical conductivity) with Fe_3_O_4_@SnO_2_ resulted in electric dipolar polarization and interfacial polarization. Hence, the composite showed an enhanced electromagnetic wave absorption effect in the high-frequency region. Chen et al. [23] fabricated Fe_3_O_4_/SnO_2_/C composite particles for application as an anode for lithium-ion batteries. The complexation of Fe_3_O_4_ and SnO_2_ improved the reversibility of the LiO_2_ conversion reaction, thereby increasing the specific capacity and charge/discharge efficiency of the anode. In addition, the surface modification of the composite with carbon improved the conductivity of the active material and its affinity with the electrolyte. Thus, the Fe_3_O_4_/SnO_2_/C composite showed a more stable rate performance than the Fe_3_O_4_/SnO_2_ particles during cycling. Therefore, the preparation of Fe_3_O_4_-SnO_2_-C composites has become a topic of great scientific interest. 

**Figure 1:**
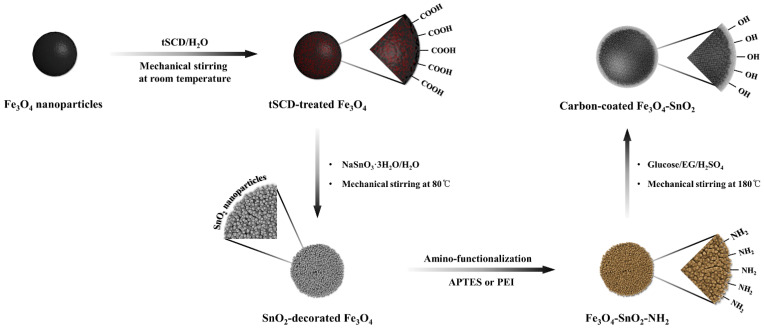
Schematic for the preparation of the Fe_3_O_4_-SnO_2_-C nanoparticles.

The fabrication of SnO_2_-decorated Fe_3_O_4_ composites through facile surface modification has been reported [24]. Fe_3_O_4_ nanoparticles were carboxylated using polyacrylic acid (PAA) or tri-sodium citrate dihydrate (tSCD), and the surface modification method using tSCD was more effective than that using PAA. The results demonstrated the feasibility of the preparation of spherical nanoparticles surrounded by ultrafine SnO_2_ nanoparticles. Motivated by these results, in this study, we prepared Fe_3_O_4_-SnO_2_-C composite particles with a three-layered core–shell structure through the dehydration of glucose under atmospheric pressure (Figure 1). Fe_3_O_4_-SnO_2_ nanoparticles were amino-functionalized using a polymeric precursor, PEI, and a silane precursor, APTES. Subsequently, the effects of the functionalizing amine groups on the structure of the multilayered particles were investigated. In addition, the mechanism underlying the formation of the core–shell structure by the reaction between the polymerized layer formed by combining glucose, a monosaccharide-based carbon precursor, and the aminated nanoparticles and sulfuric acid was investigated.

## 2. Materials and Methods

### 2.1. Materials

Ethylene glycol (EG, >99.5%, Samchun Pure Chemical, Pyeongtaek-si, Korea), ferric chloride hexahydrate (FeCl_3_·6H_2_O, >97%, Sigma Aldrich, USA), sodium acetate (NaOAc, >99.5%, Sigma Aldrich, St. Louis, MO, USA), ethanol (99.9%, Daejung Chemical, Siheung-si, Korea), tri-sodium citrate dihydrate (tSCD, HOC(COONa)(CH_2_COONa)_2_·2H_2_O, ≥99.0%, Sigma Aldrich, St. Louis, MO, USA), sodium hexahydroxostannate (Na_2_SnO_3_·3H_2_O, 95%, Junsei, Tokyo, Japan), (3-Aminopropyl)triethoxysilane (APTES, 98%, Sigma Aldrich, St. Louis, MO, USA), polyethyleneimine (PEI, branched, Mw ~25,000, Sigma Aldrich, St. Louis, MO, USA), D-(+)-glucose (>99.5%, Sigma Aldrich, St. Louis, MO, USA) and sulfuric acid (H_2_SO_4_, >98%, Daejung Chemical, Siheung-si, Korea) were used without further treatment.

### 2.2. Preparation of Core–Shell Structured Fe_3_O_4_-SnO_2_ Nanoparticles

Fe_3_O_4_-SnO_2_ nanoparticles were prepared as described in our previous report [24]. To prepare the Fe_3_O_4_ nanoparticles, 45 g of FeCl_3_·6H_2_O was added to distilled water under stirring at room temperature, and 0.5 M NaOAc was dissolved in 1000 mL of EG. These two solutions were then transferred to a three-necked round-bottom flask, and the resulting solution was heated to boiling with mechanical stirring and maintained at this temperature for 18 h. After the completion of the reaction, the solution was naturally cooled to room temperature, and the reactants were separated from the solution using a magnet. Subsequently, the reactants were rinsed with distilled water and ethanol several times to eliminate the organic and inorganic by-products.

The prepared Fe_3_O_4_ nanoparticles (1 g) were dispersed in 100 mL of distilled water. The resulting suspension was then transferred to a three-neck round-bottom flask, followed by the addition of 0.5 M tSCD. The mixed solution was mechanically stirred at 300 rpm for 24 h at room temperature. After the completion of the reaction, the surface-modified particles were separated from the solution and subsequently rinsed several times with distilled water.

The surface-modified Fe_3_O_4_ nanoparticles (0.5 g) were ultrasonically redispersed in 300 mL of distilled water for 30 min. Subsequently, 1 g of Na_2_SnO_3_·3H_2_O was added to the suspension under stirring at 300 rpm for 24 h at 80 °C. The reaction-terminated suspension was naturally cooled to room temperature, and the particles were separated using a magnet. The resultant particles were washed several times with distilled water.

### 2.3. Amino Functionalization of the Core–Shell Fe_3_O_4_-SnO_2_ Nanoparticles

The surface amino-functionalization process was carried out using two different methods. In the case of the inorganic-precursor-based amino functionalization, the Fe_3_O_4_-SnO_2_ nanoparticles (0.5 g) were ultrasonically dispersed for 15 min in a 100 mL solution of distilled water and ethanol (1:1 vol%). The dispersed Fe_3_O_4_-SnO_2_ suspension was then transferred to a three-neck round-bottom flask, followed by the injection of 0.09 M of APTES. The suspension was mechanically stirred at 350 rpm and 70 °C for 48 h. After the reaction, the solution was naturally cooled to room temperature and the reactants were separated from the solution using a magnet. The resultant particles were washed several times with ethanol and distilled water. 

In the case of the polymeric-precursor-based amino functionalization, the Fe_3_O_4_-SnO_2_ nanoparticles (0.5 g) were ultrasonically redispersed for 20 min in 200 mL of distilled water with 5 wt% of PEI. Subsequently, the dispersed suspension was mechanically stirred at 300 rpm in a round-bottom flask and heat-treated at 80 °C for 18 h. The solution was cooled to room temperature, and the resulting particles were washed with distilled water several times to eliminate the by-products. 

### 2.4. Carbonization of the Core–Shell Fe_3_O_4_-SnO_2_ Nanoparticles

The amino-functionalized Fe_3_O_4_–SnO_2_ nanoparticles (0.5 g) were redispersed in 400 mL of EG for 30 min via ultrasonication. The mixture solution was transferred to a three-neck round-bottom flask equipped with a reflux condenser, followed by the addition of 30 g of D-(+)-glucose. Subsequently, the solution was mechanically stirred at 300 rpm for 20 h at 180 °C under reflux conditions. Subsequently, the heated solution was slowly cooled to room temperature and immediately reheated to 60 °C. Then, 2 mL of 0.1 M H_2_SO_4_ was added to the solution, and the resulting solution was heated to 180 °C and maintained for 24 h. After the reaction, the solution was naturally cooled to room temperature, and the particles were separated from the solution using a magnet. The synthesized particles were washed several times with ethanol and distilled water.

### 2.5. Characterization

The surface characteristics of the surface-modified particles were investigated using Fourier transform infrared (FTIR) spectroscopy (Nicolet 5700, Thermo Electron, Waltham, MA, USA). The surface charges and dispersion properties of the particles were evaluated by carrying out zeta potential and particle size distribution analyses (Zetasizer Nano ZS, Malvern, UK). The crystal structures of the synthesized nanoparticles were analyzed using X-ray diffraction (XRD, UltimaIV, Rigaku, Japan) with Cu Kα radiation (λ = 1.5418 Å). The morphologies of the nanoparticles were investigated using high-resolution transmission electron microscopy (HRTEM, Tecnai G2 F30 S-Twin, FEI, Hillsboro, OR, USA). The degree of graphitization of the carbon layer on the particle surface was investigated using Raman spectroscopy (NRS-3100, Jasco, Easton, PN, USA), with an excitation wavelength of 532 nm. Moreover, the magnetic properties of the particles were evaluated using a vibrating sample magnetometer (Lake Shore 7400, Cryotronics Inc., Westerville, OH, USA) at the applied field of −10–10 kOe at room temperature.

## 3. Results and Discussion

**Figure 2:**
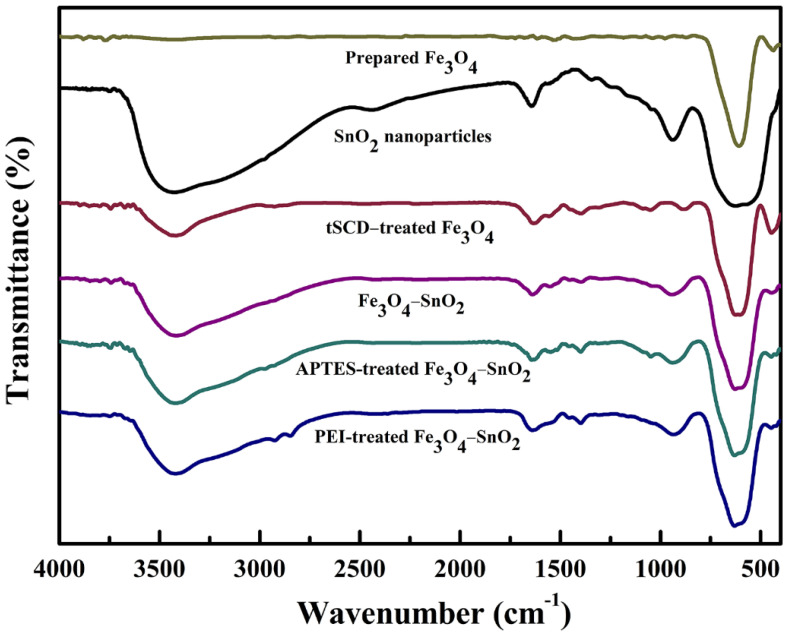
FTIR spectra of the as-prepared Fe_3_O_4_, SnO_2_, tSCD-treated Fe_3_O_4_, Fe_3_O_4_-SnO_2_, APTES-treated Fe_3_O_4_-SnO_2_, and PEI-treated Fe_3_O_4_-SnO_2_ nanoparticles.

To identify the surface functional groups of the as-prepared nanoparticles, their FTIR spectra were analyzed, as shown in Figure 2. The Fe_3_O_4_ nanoparticles exhibited a peak at 582 cm^−1^, corresponding to the stretching of the Fe-O bond in the tetrahedral sites [8]. In contrast, the SnO_2_ nanoparticles synthesized under the same experimental conditions exhibited peaks corresponding to the stretching of the Sn–OH, O–Sn–O, and Sn-O bonds at 550, 618, and 941 cm^−1^, respectively [25]. The peaks at 1639 and 3415 cm^−1^ can be ascribed to the –OH stretching vibration of the particle surface of the hydroxyl group, which was generated by the absorption of H_2_O from the ambient atmosphere [25]. The tSCD-treated Fe_3_O_4_ nanoparticles exhibited –CH_2_ and –CH_3_ vibration peaks at 2857 and 2920 cm^−1^, respectively. In addition, peaks corresponding to the vibration of the C–O and COO–Fe bonds were observed at 1393 and 1619 cm^−1^, respectively [26]. This confirms the presence of carboxyl groups on the particle surface. 

The Fe_3_O_4_-SnO_2_ nanoparticles exhibited peaks at 582 and 550 cm^−1^, corresponding to the Fe–O and O–Sn–O bonds, respectively. Additionally, the peak at 941 cm^−1^ can be ascribed to the stretching vibration of the Sn–O bond, which originated from the SnO_2_ formed on the Fe_3_O_4_ surface. In the case of the APTES-treated Fe_3_O_4_-SnO_2_ nanoparticles, an absorption peak was observed at approximately 3300 cm^−1^, corresponding to the symmetric and asymmetric stretching modes of NH/NH_2_ [27,28]. The peak at 1048 cm^−1^ can be ascribed to the Si–O–Si bond, which originated from the silane group of APTES [27]. On the other hand, the PEI-treated Fe_3_O_4_-SnO_2_ nanoparticles exhibited –CH_2_ and –CH_3_ vibration peaks at 2843 and 2919 cm^−1^, respectively [29]. In addition, symmetric and asymmetric NH/NH_2_ stretching modes were observed at 1639 and approximately 3300 cm^−1^, respectively [30]. Therefore, it can be stated that various organic functional groups were present on the surface of the PEI- and APTES-modified Fe_3_O_4_-SnO_2_ nanoparticles with different structures.

The zeta potentials of the nanoparticles were measured to examine the changes in their surface properties after their surface modification under distilled water conditions (Table 1). In the table, each sample displacement is the result of 10 measurements under the same conditions. The zeta potential value of the Fe_3_O_4_ nanoparticles was −5.4 mV, which indicates that these particles showed a small amount of surface charge. The Fe_3_O_4_-SnO_2_ nanoparticles showed a zeta potential of −42.3 mV. In contrast, the zeta potentials of both the APTES- and PEI-treated particles were positive (31.8 and 32.9, respectively) because of the formation of amine groups on their surfaces. Although these modified nanoparticles showed similar zeta potential values, the average value and standard deviation of the PEI-treated particles were slightly larger. Therefore, the initial glucose conjugation amount could be set identically by minimizing the effect of electrostatic attraction that might have been caused by the surface charge on each particle during the carbon coating process.

**Figure 3:**
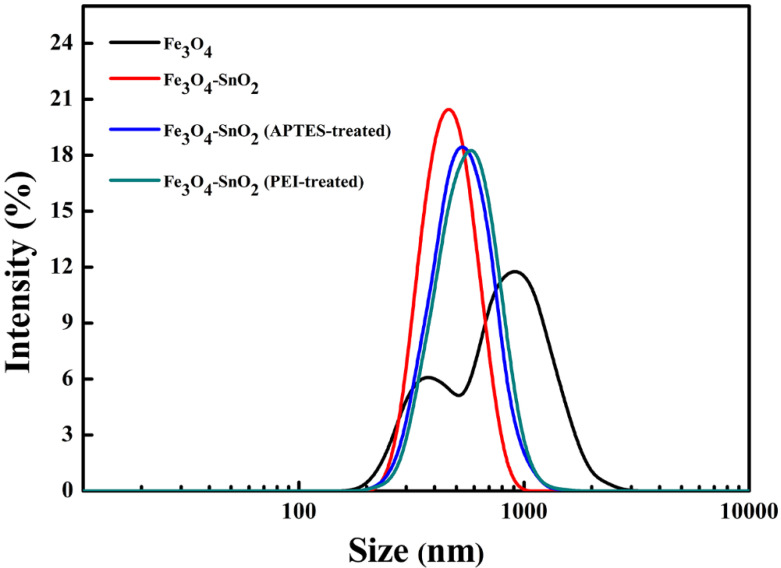
Particle size distribution curves of the as-prepared Fe_3_O_4_, Fe_3_O_4_-SnO_2_, APTES-treated Fe_3_O_4_-SnO_2_, and PEI-treated Fe_3_O_4_-SnO_2_ nanoparticles.

Figure 3 shows the particle size distributions of the as-prepared Fe_3_O_4_, Fe_3_O_4_-SnO_2_, APTES-treated Fe_3_O_4_-SnO_2_, and PEI-treated Fe_3_O_4_-SnO_2_ nanoparticles in distilled water. The distribution curve of the Fe_3_O_4_ nanoparticles showed a bimodal shape with a wide size distribution range of 200–2650 nm and a mean size of 821 nm. This can be attributed to the presence of particles aggregated because of the high surface energy generated at the nanoscale and the low surface charge of the Fe_3_O_4_ nanoparticles [31]. In contrast, the surface-decorated/functionalized nanoparticles exhibited monomodal curves. The measured mean size distribution values of the Fe_3_O_4_-SnO_2_, APTES-treated Fe_3_O_4_-SnO_2_, PEI-treated Fe_3_O_4_-SnO_2_ nanoparticles were 476, 551, and 578 nm, respectively. In the case of the Fe_3_O_4_-SnO_2_ nanoparticles, the hydroxyl groups present on the SnO_2_ surface generated a high surface charge, improving the dispersibility of the particles in the solvent. On the other hand, the APTES- and PEI-treated Fe_3_O_4_-SnO_2_ nanoparticles showed relatively low surface charge, and thus showed slightly lower dispersibility than the unfunctionalized Fe_3_O_4_-SnO_2_ nanoparticles.

**Figure 4:**
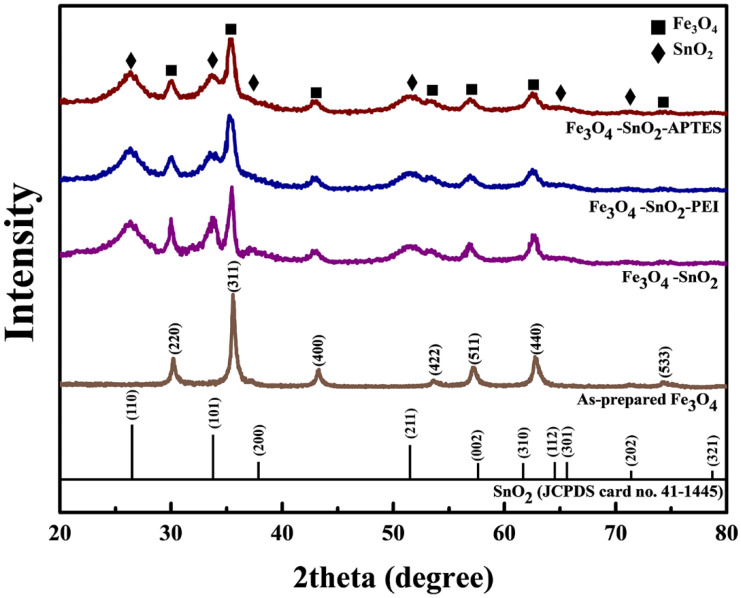
XRD patterns of the as-prepared Fe_3_O_4_, Fe_3_O_4_-SnO_2_, PEI-treated Fe_3_O_4_-SnO_2_, and APTES-treated Fe_3_O_4_-SnO_2_ nanoparticles.

The crystal structures of the Fe_3_O_4_, Fe_3_O_4_-SnO_2_, PEI-treated Fe_3_O_4_-SnO_2_, and APTES-treated Fe_3_O_4_-SnO_2_ nanoparticles were investigated using XRD (Figure 4). The Fe_3_O_4_ nanoparticles exhibited significant diffraction peaks at 2θ = 30.2, 35.5, 43.2, 53.5, 57.1, 62.7, and 74.3°, corresponding to the (220), (311), (400), (422), (511), (440), and (533) planes of the inverse-spinel structure (JCPDS No. 19-0629) [32]. The Fe_3_O_4_-SnO_2_ nanoparticles exhibited peaks corresponding to Fe_3_O_4_ in addition to those at 2θ = 26.1, 33.5, 37.3, 51.7, 65.4, and 71.7° corresponding to the (110), (101), (200), (211), (220), and (202) planes of the tetragonal rutile structure (JCPDS card no. 41-1445), respectively [33]. The diffraction peaks corresponding to SnO_2_ were broad because of its crystalline size of less than 5 nm [34]. This was further confirmed by calculating the average crystallite sizes of the prepared nanoparticles using the Scherrer’s formula, D = Kλ/βcosθ, where D is the average crystallite size, K is the shape factor (K = approximately 0.94 for spherical crystallites), λ is the X-ray wavelength (λ = 1.5418 Å for Cu K_α_ radiation), β is the full width at half maximum of the high-intensity diffraction peak (in radians), and θ is the Bragg’s angle (in radians). The average crystallite sizes of Fe_3_O_4_ and SnO_2_ were calculated to be 18.09 and 4.36 nm, respectively. Meanwhile, the XRD patterns of the APTES and PEI-treated Fe_3_O_4_-SnO_2_ nanoparticles were the same as that of the Fe_3_O_4_-SnO_2_ nanoparticles. This indicates that the Fe_3_O_4_-SnO_2_ nanoparticles maintained their crystallinity even after the amino-functionalization treatment.

**Figure 5:**
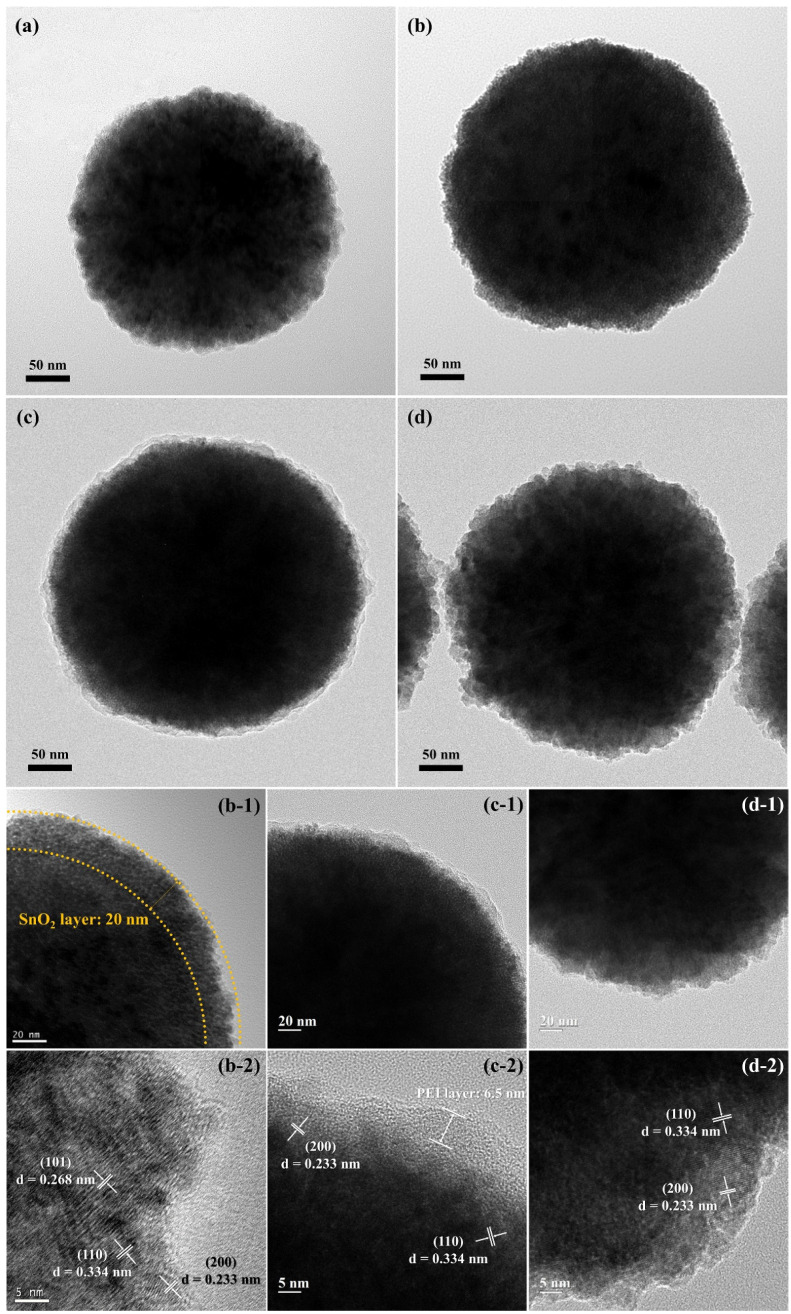
TEM and HRTEM images of the (**a**) as-prepared Fe_3_O_4_, (**b**,**b-1**,**b-2**) Fe_3_O_4_-SnO_2_, (**c**,**c-1**,**c-2**) PEI-treated Fe_3_O_4_-SnO_2_, and (**d**,**d-1**,**d-2**) APTES-treated Fe_3_O_4_-SnO_2_ nanoparticles.

Figure 5 shows the TEM and HR-TEM images showing the morphologies and microstructures of the synthesized particles before carbon coating. As shown in the figure, all the synthesized particles were spherical, and the Fe_3_O_4_ particles, which acted as the core, had a diameter of approximately 300 nm (Figure 5a). The magnified TEM (Figure 5(b-1)) and HRTEM (Figure 5(b-2)) images revealed that SnO_2_ particles with a diameter of approximately 4.5 nm were formed on the surface of the Fe_3_O_4_ particles to a thickness of approximately 20 nm. This is almost consistent with the average crystallite sizes of the Fe_3_O_4_ and SnO_2_ nanoparticles, as calculated from the XRD data (Figure 4) according to Scherrer’s formula. In addition, lattice patterns with the interplanar spacings of 0.268, 0.334, and 0.233 nm corresponding to the (101), (110), and (200) planes, respectively, were observed on the particle surface. In Figure 5(c-1), the outer layer of the PEI-treated Fe_3_O_4_-SnO_2_ nanoparticles can be clearly distinguished from that of the untreated nanoparticles. This outer layer was confirmed to be grafted onto the particle surface with a thickness of approximately 6.5 nm because of the polymerization of the polymeric precursor. In contrast, no significant difference was observed in the images of the Fe_3_O_4_-SnO_2_ nanoparticles before and after the APTES treatment (Figure 5(d-1)). Figure 5(c-2,d-2) show the HRTEM images of the PEI- and APTES-treated Fe_3_O_4_-SnO_2_ nanoparticles. Both the amine-treated nanoparticles showed lattice patterns with the interplanar spacings of 0.334 and 0.233 nm corresponding to the (110) and (200) planes of SnO_2_, respectively. This is consistent with the XRD results and confirms the successful amino functionalization of the Fe_3_O_4_-SnO_2_ nanoparticles through the polymerization of PEI or the silane bonding of APTES. However, the surface microstructures of the amino-functionalized nanoparticles prepared using these functionalization methods were significantly different.

**Figure 6:**
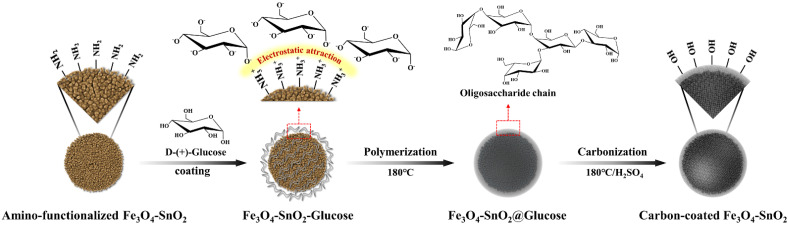
Schematic for the carbon-coating mechanism of amino-functionalized Fe_3_O_4_-SnO_2_ nanoparticles through glucose and sulfuric acid.

Figure 6 shows the carbon coating procedure of the amino-functionalized Fe_3_O_4_-SnO_2_ nanoparticles using glucose. EG, which was used as the solvent, played an important role in forming a stable dispersion of the reaction medium and functionalized magnetic nanoparticles. First, owing to its negative charge, the hydroxyl group of glucose bonded strongly to the amine group (with a positive charge) on the surface of the Fe_3_O_4_-SnO_2_ particles by electrostatic attraction. Glucose transformed into oligosaccharide chains by the condensation reaction and surrounded the particle surface. In addition, the rotational energy generated by stirring facilitated the deposition of a uniform layer of the oligosaccharide chains on the surface of the particles. Then, the added sulfuric acid dehydrated the oligosaccharide chains bonded to the surface, and a hydrophilic carbon layer with a hydroxyl group at the end was finally formed.

**Figure 7:**
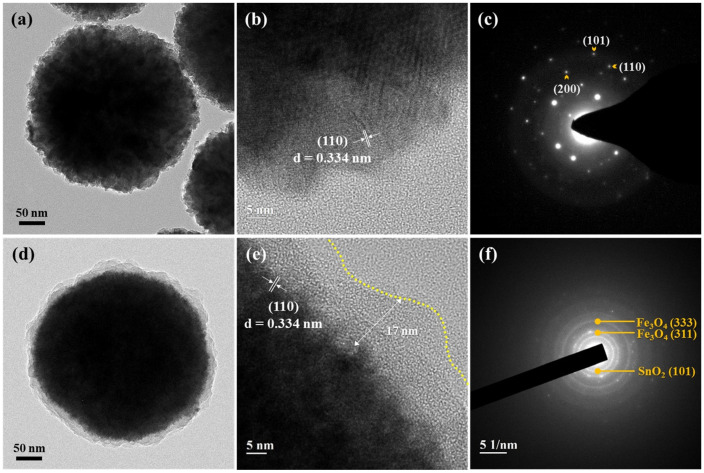
HRTEM images and SAED patterns of (**a**–**c**) the PEI-treated Fe_3_O_4_-SnO_2_ and (**d**–**f**) APTES-treated Fe_3_O_4_-SnO_2_ nanoparticles after the carbon coating process.

The TEM images of the amino-functionalized nanoparticles subjected to the carbon coating process are shown in Figure 7. In the case of the PEI-treated nanoparticles, only naked nanoparticles with the polymer layer removed were observed after the carbon coating process (Figure 7a). The polymer layer disappeared because of dissolution by high temperature or sulfuric acid during the reaction. Since PEI, as a binding polymeric material, has a melting point of approximately 75 °C, it is sensitive to heat and acidic environments [35]. However, it seems that the SnO_2_ particles formed on the surface of the Fe_3_O_4_ particles by electrostatic attraction were not removed by sulfuric acid (Figure 7b). To analyze the structure of the nanoparticles in detail, their selected area electron diffraction (SAED) patterns were recorded, which confirmed the presence of the (101), (110), and (200) planes of SnO_2_ in the composite nanoparticles (Figure 7c) [36]. 

In contrast, the particles treated with APTES formed a new coating layer on the surface with a thickness of approximately 17 nm (Figure 7d,e). In addition, the lattice pattern corresponding to the (110) plane was observed with an interplanar spacing of 0.334 nm in the HRTEM image. Ring patterns corresponding to the (333) and (311) planes of Fe_3_O_4_ and the (101) plane of SnO_2_ were observed, as shown in Figure 7f [29]. This can be attributed to the carbonization of the nanoparticles after their electrostatic bonding with glucose because the amino-functional group formed at the end of the particles by the silane bond of APTES was resistant to high temperatures and acidic environments. Therefore, it can be stated that the formation of the carbon layer was directly related to the thermal/chemical stability of the amine retention layer, and the surface treatment using APTES was more effective in carbon layer formation than that using the polymeric precursor, PEI. Finally, multilayered core–shell Fe_3_O_4_-SnO_2_-C nanoparticles were synthesized by the amino functionalization of the Fe_3_O_4_-SnO_2_ particles through APTES.

**Figure 8:**
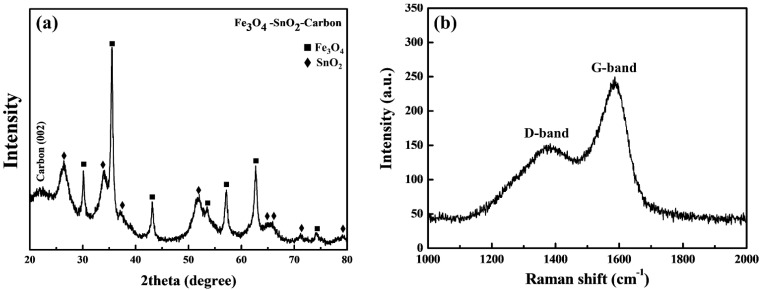
XRD patterns (**a**) and Raman spectra (**b**) of the Fe_3_O_4_-SnO_2_-C composite nanoparticles.

Figure 8a shows the XRD pattern of the Fe_3_O_4_-SnO_2_-C composite nanoparticles. The nanoparticles showed peaks of Fe_3_O_4_ and SnO_2_ corresponding to the (110), (101), (200), (211), (220), (202), (220), (311), (400), (422), (511), and (440) planes. In addition, the low-intensity peak at 2θ = 22.1° corresponds to the (002) plane of carbon, which originated from the carbon layer formed by the carbonization of glucose [37]. 

As shown in Figure 8b, the Raman spectrum of the Fe_3_O_4_-SnO_2_-C composite nanoparticles confirmed the formation of carbon on their surface. Two strong characteristic carbon peaks were observed over the wavenumber range of 1000–2800 cm^−1^. In general, the D band, which appears only when disordered or finite-sized graphite crystals are present, is not identified in well-crystallized graphite [38]. The D band at 1371 cm^−1^ indicates the presence of sp^3^ defects in the carbon network, whereas the G band at 1584 cm^−1^ corresponds to the E_2g_ stretching vibration of graphite with sp^2^ electronic configuration. 

The intensity ratio of the D- and G-bands (I_D_/I_G_) provides useful information about the crystallinity of carbon material. The closer the I_D_/I_G_ value to zero, the higher is the degree of structural alignment of the formed carbon material [39]. The I_D_/I_G_ ratio of the Fe_3_O_4_-SnO_2_-C composite nanoparticles was approximately 0.5706, as determined using Lorentz fitting. This indicates that the formed carbon layer had a fairly ordered structure. However, this carbon layer can be considered amorphous carbon because of its high I_D_ value. Thus, the layer formed on the surface of the Fe_3_O_4_-SnO_2_ nanoparticles was confirmed to be amorphous graphitized carbon, which was formed when the sulfuric acid used in the coating process promoted the dehydration reaction of the oligosaccharide obtained from glucose. Thus, the TEM and Raman analysis results indicate that the Fe_3_O_4_-SnO_2_-C nanoparticles were synthesized by the formation of a structurally aligned carbon layer in the reflux process under atmospheric pressure.

**Figure 9:**
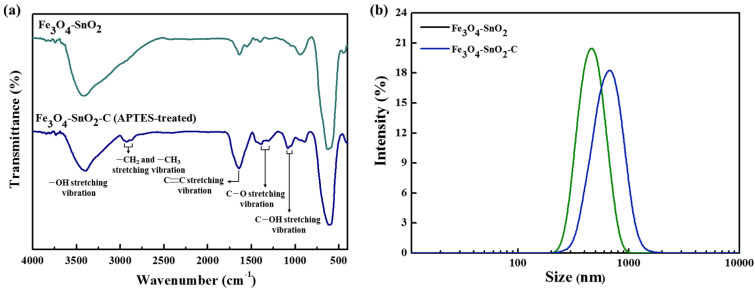
FTIR spectra (**a**) and particle size distribution curves (**b**) of the Fe_3_O_4_-SnO_2_ and Fe_3_O_4_-SnO_2_-C nanoparticles.

Figure 9a shows the FTIR spectra of the Fe_3_O_4_–SnO_2_ and Fe_3_O_4_–SnO_2_–C nanocomposites. Unlike Fe_3_O_4_-SnO_2_, the Fe_3_O_4_-SnO_2_-C nanocomposites exhibited a –OH stretching vibration peak over a broad spectral region of 3630–3200 cm^−1^. The peaks at 2917 and 2871 cm^−1^ correspond to the vibration of the –CH_2_ and –CH_3_ groups, respectively. The strong peak at 1636 cm^−1^ can be ascribed to the C–C stretching vibration. In addition, the characteristic peaks at approximately 1387–1301 and 1042–1083 cm^−1^ correspond to the stretching vibration of the C–O and C–OH bonds, respectively, which were probably formed because of the hydroxyl group formed on the carbon layer [40]. As a result, the Fe_3_O_4_-SnO_2_-C nanoparticles exhibited high hydrophilicity because of the large number of hydroxyl groups present in the carbon coating layer.

Figure 9b shows the size distribution curves of the Fe_3_O_4_-SnO_2_-C nanoparticles. As can be observed, the estimated mean size of the particles was 669 nm, and their size distribution was in the range of 225–1700 nm. As compared with the Fe_3_O_4_-SnO_2_ nanoparticles (mean size of 476 nm with a zeta potential of −42.3 mV), the Fe_3_O_4_-SnO_2_-C nanoparticles (with a higher zeta potential of −51 mV) showed aggregation. This can be attributed to the carbonization of the Fe_3_O_4_-SnO_2_ nanoparticles in a bonded state between the oligosaccharide coating layers during the carbon coating process. Nevertheless, the Fe_3_O_4_-SnO_2_-C nanoparticles showed high dispersibility in aqueous solvents because of the hydroxyl groups present on the surface of the carbon layer.

**Figure 10:**
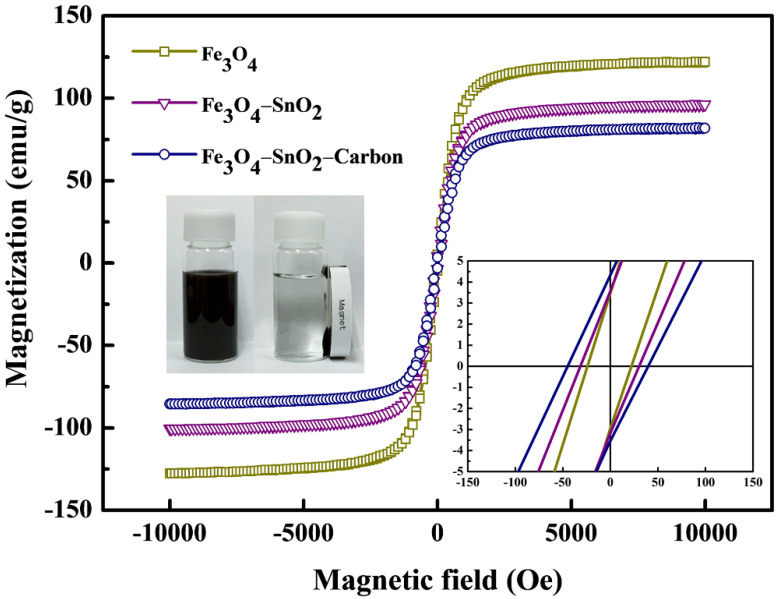
Magnetization curves of the Fe_3_O_4_, Fe_3_O_4_-SnO_2_, and Fe_3_O_4_-SnO_2_-C nanoparticles at room temperature.

As can be observed from the lower right inset of Figure 10, the coercivity (Hc) and residual magnetization (Mr) of the Fe_3_O_4_, Fe_3_O_4_-SnO_2_, and Fe_3_O_4_-SnO_2_-C nanoparticles were almost zero. This suggests that all the prepared nanoparticles were superparamagnetic. As the prepared Fe_3_O_4_ particles gradually evolved into the Fe_3_O_4_-SnO_2_-C particles, their saturation magnetization (M_S_) value tended to decrease. The measured M_S_ values of the Fe_3_O_4_, Fe_3_O_4_-SnO_2,_ and Fe_3_O_4_-SnO_2_-C nanoparticles were 121, 95, and 81 emu/g, respectively (Figure 10). This decrease in the M_S_ of the Fe_3_O_4_-SnO_2_-C nanoparticles can be attributed to the increase in the weight of the single particles because of the formation of non-magnetic SnO_2_ and carbon in the Fe_3_O_4_ nanoparticles. As can be seen from the optical image inserted, the Fe_3_O_4_-SnO_2_-C nanoparticles reacted strongly to the external magnetic fields despite their relatively lower magnetization than that of the Fe_3_O_4_-SnO_2_ nanoparticles.

## 4. Conclusions

Multilayered core–shell-structured Fe_3_O_4_-SnO_2_-C nanoparticles were fabricated via surface treatment and carbonization at atmospheric pressure. The carboxylation of Fe_3_O_4_ nanoparticles using tSCD fixed SnO_2_ nanoparticles electrostatically on them, with a mean size of 4.5 nm while maintaining a layer thickness of 20 nm. Thereafter, the surface amino functionalization of the Fe_3_O_4_-SnO_2_ nanoparticles using PEI and APTES was carried out at the hydroxyl group at the end of the glucose molecule by electrostatic attraction. Unfortunately, when PEI was used, the delicate polymer layer was destroyed by high temperatures and sulfuric acid during the carbon coating process, and the final product was Fe_3_O_4_-SnO_2_. Therefore, the polymer-based amino functionalization was not suitable for the carbonization process. When APTES was used, the amine group formed on the surface of the nanoparticles by silane bonding facilitated the formation of a carbon layer even under severe coating conditions. The carbonized layer formed by the dehydration of glucose with sulfuric acid was hydrophilic amorphous graphitized carbon. The Fe_3_O_4_-SnO_2_-C nanoparticles showed an M_S_ of approximately 81 emu/g and showed excellent separation performance, which is typical of superparamagnetic nanoparticles.

## Figures and Tables

**Table 1:** Zeta potentials of the as-prepared Fe_3_O_4_, Fe_3_O_4_-SnO_2_, APTES-treated Fe_3_O_4_-SnO_2_, and PEI-treated Fe_3_O_4_-SnO_2_ nanoparticles in the presence of distilled water.

Sample	Zeta Potential (mV)	Standard Deviation
Prepared Fe_3_O_4_	−5.4	0.08
Fe_3_O_4_-SnO_2_	−42.3	1.17
APTES-treated Fe_3_O_4_-SnO_2_	31.8	1.05
PEI-treated Fe_3_O_4_-SnO_2_	32.9	1.68

## Data Availability

All data used to support the findings of this study are included in this article.
